# Acute Myocardial Infarction Secondary to Catecholamine Release Owing to Cocaine Abuse and Pheochromocytoma Crisis

**DOI:** 10.5812/ijem.6562

**Published:** 2012-12-21

**Authors:** Efren Martinez-Quintana, Ricardo Jaimes-Vivas, Javiel Cuba-Herrera, Beatriz Saiz-Udaeta, Fayna Rodríguez-Gonzalez, Maria Soledad Martinez-Martin

**Affiliations:** 1 Cardiology Service, Insular-Materno Infantil University Hospital, Las Palmas de Gran Canaria, Spain; 2 Ophtalmology Service, Dr. Negrin University Hospital of Gran Canaria, Las Palmas de Gran Canaria, Spain; 3 Anatomopathology Service, Insular-Materno Infantil University Hospital, Las Palmas de Gran Canaria, Spain

**Keywords:** Pheochromocytoma, Cocaine, Myocardial Infarction

## Abstract

**Abstract:**

Most pheochromocytomas are not suspected clinically while a high percentage of them are curable with surgery. We present the case of an adult cocaine-addicted male patient with an underlying pheochromocytoma and repeated myocardial infarctions. Computed tomography showed a left round adrenal mass, also high 24-hour urine levels of catecholamines and metanephrines were detected from urinalysis. The patient was given alpha and beta blockers, moreover a laparoscopic left adrenalectomy was performed. Cocaine can block the reuptake of noradrenaline, leading to increasing its concentration and consequently its effects as well, and induce local or diffuse coronary vasoconstriction in normal coronary artery segments per se, cocaine can also trigger pheochromocytoma crisis, and therefore, cardiac complications such as myocardial infarction due to these additive effects are intended to occur. For this reason, in the presence of typical clinical manifestations of pheochromocytoma, such as sustained or paroxysmal hypertension, headache, sweating, tachycardia and abdominal pain, probable association of this tumor in patients with cocaine abuse and associated cardiac complications must be ruled out.

## 1. Introduction

The pheochromocytoma is a catecholamine secreting tumour derived from chromaffin cells of the sympathetic nervous system. Eighty to eighty-five percent of these tumours are localized in the adrenal medulla but when pheocromocytomas are found outside the adrenal gland they are referred to as extra-adrenal pheochromocytomas or paragangliomas (1). Pheochromocytomas can occur in patients of any age, but increases with advancing age. Its incidence is similar in both genders. It is estimated that 1 to 8 cases of pheochromocytoma occur per million annually (2).

Typical clinical manifestations include sustained or paroxysmal hypertension caused by intermittent release of these hormones, and severe headaches, palpitations and sweating resulting from hormone excess. However, their presentation is highly variable and can mimic many other diseases (3). Most of the life-threatening cardiovascular manifestations of pheochromocytoma, such as hypertensive emergencies, result from a rapid and massive release of catecholamines from the tumor which can be induced by increased abdominal pressure, trauma, surgery, stress, exercise, ingestion of foods or beverages containing tyramine such as certain cheeses, beers, and wines or consumption of certain drugs such as histamine, glucagon, tyramine, phenothiazine, metoclopramide, adrenocorticotropic hormone or cocaine (4). The diagnosis is confirmed by elevation of catecholamines and the metanephrines in blood plasma and urine. Localization of the tumour should be done following biochemical diagnosis by means of computed tomography (CT) scan and/or magnetic resonance imaging (MRI) , being the treatment of choice the resection of the tumor by laparoscopic surgery (1).

The aim of this report is to show how sympathomimetic agents such as cocaine can trigger pheochromocytoma crisis and explain its possible mechanisms by which cocaine exacerbates the course of the disease.

## 2. Case Report

A 41-year-old male patient with a medical history of dyslipidemia, cocaine abuse and two previous non ST elevation myocardial infarctions with normal findings of coronary arteries angiography, admitted to the emergency department due to an intense abdominal pain of sudden onset. The patient has referred during the last months, a history of pallor, palpitations, tremors, sweating, and severe abdominal pain (1-2 times per week) more predominantly in the mornings and with alcohol intake. Clinical examinations showed arterial hypertension, sinus tachycardia, fever and facial flushing. No murmurs were heard. Abdomen was soft, depressible and painful on palpation (mild pain) in both hypochondria, predominantly in the left flank. The electrocardiogram showed sinus tachycardia at 120 beats per minute and diffuse ST-segment depression. Blood analysis results showed leukocytosis (34,700/µL), renal insufficiency (serum creatinine: 3.58 mg/dL), metabolic acidosis, high serum lactate levels (14 mM/L) and high cardiac enzymes (maximum troponin detected of 4.43 ng/mL with a creatine phosphokinase (CPK) of 3967 U/L). Echocardiography showed no segmental left ventricular wall motion abnormalities. Findings of chest and abdominal radiography were normal. Urine toxic screen result was positive for cocaine.

**Figure 1:**
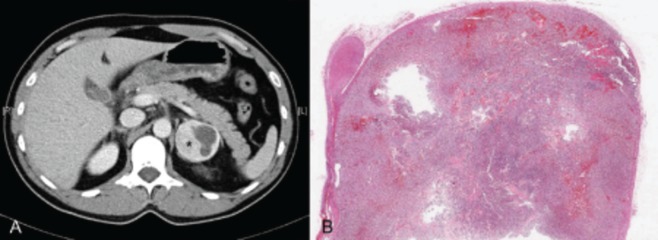
Computed Tomography and Histological Images of the Pheochromocytoma A: Abdominal computed tomography showing a left adrenal mass of 50 mm in diameter with rounded, well-defined edges, and hyperdense areas of cystic necrosis inside (asterisk). B: Histological panoramic view of the pheochromocitoma. On the left side of the picture there is a normal adrenal gland on which sits the tumor with a large nodule with areas of hemorrhagic aspect, especially in the tumor periphery.

Despite having the diagnosis of non Q myocardial infarction secondary to cocaine abuse, due to persistent abdominal pain, abdominal CT was performed, showing a left round adrenal mass of 50 mm diameter, well-defined edges and hyperdense areas of cystic necrosis inside (Figure 1A). 24-hour urine catecholamine collection showed high levels of noradrenaline [3542 mcg/24h (NV: 0-76)] and adrenaline [1454 mcg/24h (NV: 0-18.0)] with normal dopamine levels [304 mcg/24h (NV: 0-390)]. Similarly, measurement of 24-hour urine metanephrines levels showed high metanephrine [21547.0 mcg/24h (NV: 0-341)] and normetanephrine [10729.0 mcg/24h (NV: 88-444)] concentrations.

The patient was given alpha (phenoxybenzamine 20 mg/12hours) and beta (propranolol 20 mg/6hours) blockers; also a laparoscopic left adrenalectomy was performed. Histology evaluation confirmed the presence of a pheochromocytoma without capsular invasion and a low mitotic index (Figure 1B).

## 3. Discussion

Pheochromocytomas are catecholamine-producing tumors (usually noradrenaline) derived from the sympathetic or parasympathetic nervous system. Cardiac symptoms are variable, ranging from asymptomatic forms seen in patients with adrenal incidentalomas to patients with hypertensive crisis (4), ST elevation (5) and non-ST elevation (6) acute myocardial infarction, tako-tsubo cardiomyopathy (7), adrenergic myocarditis (8), cardiomyopathies (9), QT prolongation (10), ventricular tachycardia (11) or cardiac heart failure (12). In fact, Yu et al.(13) showed that 12% of patients with pheochromocytoma had cardiac complications, additionally, focal myocardial necrosis and inflammation were present in 50% of patients who died of pheochromocytoma (8). This paradigm was further supported by reports whereby transient cardiomyopathy has originated from the exogenous administration of catecholamines such as after intravenous beta agonist administration for asthma treatment (14) or subcutaneous administration of adrenaline in life-threatening situations (15).

Cocaine blocks the reuptake of noradrenaline and dopamine at preganglionic synaptic nerve endings, increasing the synaptic concentrations of these monoamines, therefore enhancing the effects of noradrenaline. Release of noradrenaline from adrenergic nerve terminals has been shown to cause diffuse and focal occlusive vasospasm in patients with normal coronary arteries (16-18). However, other pathophysiologic mechanisms such as increased myocardial contractility, heart rate and blood pressure, endothelial dysfunction and appearance of coronary thrombosis also appears to contribute to myocardial ischemia (19) in cocaine-addicted patients. In fact, there is a 24-fold increased risk of acute myocardial infarction during the initial 60 minutes after cocaine consumption in patients who are otherwise at low risk (17), and myocardial infarction has been reported in 0.7% to 6% of patients who have had chest pain after cocaine consumption (20). Similarly, many conditions such as tricyclic antidepressant consumption, drugs stimulating adrenergic receptors, dopamine antagonists, many drugs prescribed for obesity and other over the counter medications such as nasal decongestants containing ephedrine, pseudoephedrine, or phenylproanolamine can also precipitate myocardial infarction in individuals with underlying pheochromocytomas (21).

The classic triad of pheochromocytoma presentation is episodic headache, sweating, and palpitations (22), pheochromocytoma is typically presented with a diverse set of symptoms, which may include anxiety, chest and abdominal pain, visual blurring, papilledema, nausea and vomiting, transient electrocardiographic changes, and psychiatric disorders (21, 23) some of which can also be seen in cocaine-addicted patients. If we take into account the role of cocaine in releasing noradrenaline and adrenaline from the adrenal medulla (24), in addition to many clinical conditions and types of stress such as myocardial infarction and congestive heart failure which may elevate plasma and urinary catecholamines and their metabolites, the diagnosis of pheochromocytoma can be difficult in cocaine-addicted patients with cardiac complications.

The biochemical diagnosis of pheochromocytoma depends on the demonstration of excessive production of catecholamines. Metanephrines measurement is more sensitive than the catecholamines because of their higher stability. Although CT and MRI have excellent sensitivity in detecting most catecholamine-producing tumors, these anatomical imaging approaches lack the specificity required to unequivocally identify a mass as a pheochromocytoma or paraganglioma (25), necessitate the need for a biopsy or the adrenalectomy for an histological diagnosis. During surgical manipulation of the tumor, massive catecholamine release may occur. This can result in hypertensive crisis, cardiac arrhythmias, cerebrovascular accidents, myocardial infarction or ischemia, pulmonary edema, and consequently multiorgan failure. For this reason, pharmacological pretreatment is essential to reduce the morbidity and mortality rate from as high as 45% to less than 2% (26).

Though cocaine can induce local or diffuse coronary vasoconstriction in normal coronary artery segments, the combination of cocaine and pheochromocytoma may have an additive effect reflected in an increased risk of cardiac complications such as myocardial infarction. For this reason, in cocaine-addicted patients with repetitive cardiac complications we should take into account the clinical of pheochromocytoma to rule it out.
